# Gut to brain interaction in Autism Spectrum Disorders: a randomized controlled trial on the role of probiotics on clinical, biochemical and neurophysiological parameters

**DOI:** 10.1186/s12888-016-0887-5

**Published:** 2016-06-04

**Authors:** Elisa Santocchi, Letizia Guiducci, Francesca Fulceri, Lucia Billeci, Emma Buzzigoli, Fabio Apicella, Sara Calderoni, Enzo Grossi, Maria Aurora Morales, Filippo Muratori

**Affiliations:** IRCCS Stella Maris Foundation, Viale del Tirreno 331, 56018 Calambrone, Pisa, Italy; National Research Council, Institute of Clinical Physiology, Via Moruzzi 1, Pisa, 56124 Italy; Department of Clinical and Experimental Medicine, University of Pisa, Via Savi, 10, 56126 Pisa, Italy; Department of Autism Research, Villa Santa Maria Institute, Via IV Novembre 15 22038, Tavernerio, Italy

**Keywords:** Autism Spectrum Disorders (ASD), Gut-brain axis, Endophenotype, Probiotic Vivomixx®, Quantitative electroencephalography (QEEG), Phtalates

## Abstract

**Background:**

A high prevalence of a variety of gastrointestinal (GI) symptoms is frequently reported in patients with Autism Spectrum Disorders (ASD). The GI disturbances in ASD might be linked to gut dysbiosis representing the observable phenotype of a “gut-brain axis” disruption. The exploitation of strategies which can restore normal gut microbiota and reduce the gut production and absorption of toxins, such as probiotics addition/supplementation in a diet, may represent a non-pharmacological option in the treatment of GI disturbances in ASD. The aim of this randomized controlled trial is to determine the effects of supplementation with a probiotic mixture (Vivomixx®) in ASD children not only on specific GI symptoms, but also on the core deficits of the disorder, on cognitive and language development, and on brain function and connectivity. An ancillary aim is to evaluate possible effects of probiotic supplementation on urinary concentrations of phthalates (chemical pollutants) which have been previously linked to ASD.

**Methods:**

A group of 100 preschoolers with ASD will be classified as belonging to a GI group or to a Non-GI (NGI) group on the basis of a symptom severity index specific to GI disorders. In order to obtain four arms, subjects belonging to the two groups (GI and NGI) will be blind randomized 1:1 to regular diet with probiotics or with placebo for 6 months. All participants will be assessed at baseline, after three months and after six months from baseline in order to evaluate the possible changes in: (1) GI symptoms; (2) autism symptoms severity; (3) affective and behavioral comorbid symptoms; (4) plasmatic, urinary and fecal biomarkers related to abnormal intestinal function; (5) neurophysiological patterns.

**Discussion:**

The effects of treatments with probiotics on children with ASD need to be evaluated through rigorous controlled trials. Examining the impact of probiotics not only on clinical but also on neurophysiological patterns, the current trial sets out to provide new insights into the gut-brain connection in ASD patients. Moreover, results could add information to the relationship between phthalates levels, clinical features and neurophysiological patterns in ASD.

**Trial registration:**

ClinicalTrials.gov Identifier: NCT02708901. Retrospectively registered: March 4, 2016.

## Background

Autism Spectrum Disorders (ASD) comprise a complex group of disorders of brain development characterized by social and communication impairment along with presence of repetitive and restrictive behaviors [1]. An elevated prevalence of gastrointestinal (GI) dysfunction in individuals with ASD was first reported by Goodwin and colleagues in 1971 [2], and repeatedly confirmed in subsequent studies [3–9], with prevalence rates ranging from 9 to 70 % [7, 10]. The most frequently reported GI symptoms were alterations in bowel habits, chronic abdominal pain, reflux and vomiting [8, 11–13]. It is notable that untreated GI symptoms may increase behavioral problems in children with ASD [4, 14]. Several studies reported also dysbiosis or various altered composition of intestinal microbiota in ASD children [15–18]. In particular, a greater representation of members of the family of Clostridiales [16–18] and an increase of Sutterella and Ruminococcus torques populations [19] has been repeatedly revealed, the so called “gut-brain axis” has been described as a physiological bidirectional complex network of communication between the brain and the gut [20–22] and it has been suggested that this network is involved in neurodevelopment [23–29] as well as in a variety of neuropsychiatric diseases [30] including ASD [5, 22, 31, 32]. Therefore, the GI disturbances in ASD might be linked to gut dysbiosis representing the observable phenotype of a “gut-brain axis” disruption [8, 15, 33, 34] characterized by intestinal inflammation and alterations in intestinal function, often described as a "leaky gut" [35].

Among the potential mechanisms by which gut microbiota can affect Central Nervous System (CNS) function, there are the indirect effects on the innate immune system through circulating levels of pro-inflammatory and anti-inflammatory cytokines or through production of metabolites (e.g. short chain fatty acids –SCFAs-) that modulate the immune system and the sympathetic nervous system [35]. In particular, changes in the gut microbiota are thought to be related to reduced integrity of the intestinal barrier, leading to increased absorption of toxins from the gut lumen and leakage of lipopolysaccharides (LPS) and fatty acids. These molecules can act upon Toll-like receptor 4 to activate systemic inflammation [36] which, in turn, affects critically also the CNS. Growing evidence indicates that ASD pathogenesis may involve brain inflammation associated with increased inflammatory biomarkers, such as Interleukin-6 (IL-6) and Tumor Necrosis Factor-α (TNF-α) [36] and recent studies have demonstrated that cytokines (in particular adipokines) are the key molecules responsible for the immune cell interaction. According to this view, Rodrigues and colleagues [37] have found decreased levels of resistin and increased levels of leptin in plasma of ASD subjects in comparison with controls.

Another potential and noninvasive marker of intestinal inflammation recently studied in ASD is calprotectin [38], an abundant neutrophil protein found in the stool that is markedly elevated in infectious and inflammatory conditions, including Inflammatory Bowel Disease (IBD).

Other “gut-brain” interaction mechanisms are bidirectional signalling via the vagal nerve, dysregulation of metabolic pathways involved in production of neurotransmitters (such as serotonin and Gamma-Amino Butyric Acid), and direct effects on the release of gut peptides with modulatory functions from enteroendocrine cells [35].

All these mechanisms could interfere with brain development and function, contributing to the pathogenesis of ASD through modulation of brain neurotransmission and connectivity, both at local and global level. In particular, an alteration in brain connectivity is one of the strongly supported neural underpinning of ASD symptoms [39, 40]. Recently, Quantitative Electroencephalography (QEEG), a non-invasive technique that allows a highly precise measurement of brain function and connectivity, has been applied to link neurophysiological patterns with ASD symptoms, in order to characterize ASD subgroups and to evaluate the effect of treatment [41].

The research interest on GI problems in ASD is therefore based on its practical therapeutic importance as well as on its theoretical significance. Indeed, it has been suggested that both GI disturbances and altered gut microflora could make a child with a genetic predisposition for ASD more prone to express the autistic phenotype or increase the severity of the autistic behavior [17, 34, 42]. If this hypothesis was true then it would be plausible to expect that strategies aimed at restoring normal gut microbiota, such as probiotics supplementation in the diet, may represent a non-pharmacological option not only for GI disturbances [43], but also for behavioral and neurophysiological abnormalities associated with ASD.

Probiotics are live microorganisms thought able to stabilize the mucosal barrier by increasing mucin expression, reducing bacterial over growth, stimulating mucosal immunity (secretory Immunoglobulin A), and synthesizing antioxidant substances [32, 44]. Accordingly to their balancing role on the intestinal microbiota, and to their anti-proliferative activity on Clostridium species [45–49], probiotics may represent a treatment in disorders in which dysbiosis and increased intestinal permeability have been reported, including ASD [32, 50].

Moreover, a further possible effect of probiotic supplementation could be related to a reduction of metabolites of exogenous toxic substances, through an indirect effect on their absorption by the “leaky gut”. After all, research studies strongly support a significant contribution of environmental factors in addition to genetic factors in ASD etiology [51]. A class of chemical pollutants which has been recently investigated in ASD is represented by phthalates, chemicals widely used as plasticizers, solvents and additives in many consumer products [52, 53].

To our knowledge, four different studies explored the effects of probiotics in ASD [34, 54–56], but none of them was designed or powered to assess significant differences in clinical outcomes or identify adverse effects [57]. Although all aforementioned studies showed changes in gut flora after the implementation diet with probiotics, only some of them analyzed the correlations with GI symptoms [34, 56].

Among these four studies [34, 54–56], only Parracho et al. [54] detected significant behavioral changes in ASD patients treated with probiotics compared to ASD individuals treated with placebo. Kaluzna-Czaplinska and colleagues [55] found significant metabolic modifications in ASD after probiotic administration. Two investigations limited their findings to a strong positive correlation between autism severity and the severity of GI dysfunction [34, 56] without analyzing the effects of probiotics on ASD clinical features. Although encouraging and supported by more robust preclinical evidence [50], data on beneficial GI and behavioral effects in ASD populations after probiotic treatment needs yet to be established in well-controlled clinical trials [57, 58].

### Objectives (Research aims and hypothesis)

Children with ASD and GI symptoms could represent a distinct ASD endophenotype with specific clinical and neurophysiological features. The exploitation of strategies which can restore normal gut microbiota, and reduce the gut production and absorption of toxins, such as probiotics, may represent a non-pharmacological option in the treatment of GI disturbances in ASD. Moreover, a treatment with probiotics in this subgroup of children with ASD, acting on the gut microflora and on the systemic inflammatory status, could modify their brain activity and function, possibly leading to significant improvement of their behavioral and developmental profiles.

The main aim of this study is to determine the effects of supplementation with a probiotic mixture (Vivomixx®) in ASD children with or without GI symptom. In particular, modifications on ASD core deficits, on cognitive and language development, and on specific GI symptoms will be assessed. The concomitant changes that are associated with administered treatment will be evaluated on a cluster of plasmatic, urinary and fecal biomarkers related to abnormal intestinal function as well as on neurophysiological patterns.

An ancillary aim of the study will be to determine the environmental exposure to phthalates (chemical pollutant) in ASD children, and to evaluate the possible effects of probiotics on their urinary concentrations.

As for the trial design, the study will be a double-blind randomized controlled trial with a nutritional supplement, with four parallel arms, an allocation ratio of 1:1, and a superiority framework.

## Methods/Design

### Participants

We aim to enroll 100 preschoolers with ASD who will be recruited from the Child and Adolescence Mental Health Services of Tuscany Region, and from the Unit of Child Psychiatry and the Unit of Child Rehabilitation of IRCCS Stella Maris Foundation (Pisa, Italy). This is a tertiary care University Hospital with a unit, exclusively dedicated to ASD, who receives approximately 250 patients per year (mostly preschool children) for diagnosis and treatment from all over Italy. All children recruited will be assessed at IRCCS Stella Maris Foundation.

The following inclusion and exclusion criteria will be adopted:

Inclusion criteria

- age-range: 18–72 months. The interest in assessing the effects of probiotic supplementation in preschoolers with ASD relies on the strong recommendation to lead early treatments in ASD, given the demonstrated positive impact of early interventions on the core symptoms of the disorder [59].
- ASD diagnosis according to DSM-5 (Diagnostic and Statistical Manual of Mental Disorders-5th Edition) criteria [1]: Participants must have a diagnosis of an ASD as assessed by a senior child psychiatrist with a specific expertise in clinical evaluation of ASD according to DSM-5 criteria before their recruitment in the study.

Exclusion criteria

a. brain anomalies detected by Magnetic Resonance Imaging; b. neurological syndromes or focal neurological signs; c. anamnesis of birth asphyxia, severe premature birth (≤28 gestational weeks) or perinatal injuries; d. epilepsy; e. significant sensory impairment (e.g., blindness, deafness); f. diagnosis of organic GI Disorder (i.e. gastro-esophageal reflux, food allergies, IBD); g. diagnosis of Coeliac Disease; h. special diet (i.e. gluten-free diet, casein-free diet, high-protein diet, ketogenic diet).

The criteria from a. to e. are intended to exclude children affected by not idiopathic forms of ASD or by particular endophenotypes of the disorder as the “ASD plus epilepsy” one. The criteria f. and g. are intended to exclude children with not idiopathic or not functional GI disorders. Children already enrolled in dietetic treatment studies will not be included (criteria h.) given the possible interferences of such diets on GI system during the probiotic/placebo supplementation.

### Baseline assessment

At the time of enrollment (Time 0 - T0), each research participant will undergo a comprehensive clinical, biochemical and electrophysiological evaluation in order to establish the baseline of the primary and secondary outcome measures (Table 1).

**Table 1:** Scheme of the study

TIMEPOINT of visit/assessment	Baseline	15 days (call interview)	3 months	6 months- end of the study
Enrollment				
Information and informed consent	✓			
Randomization-allocation	✓			
Interventions				
Probiotic Intervention Placebo Intervention				
Assessment				
Physical examination including anthropometric measures and abdominal tenderness	✓		✓	✓
Drug compliance and adverse event assessment		✓	✓	✓
Weekly food diaries			✓	✓
ADI-R	✓			
Outcome measures				
ADOS 2	✓			✓
CARS	✓			✓
RBS-R	✓		✓	✓
Sensory Profile	✓		✓	✓
SCQ	✓		✓	✓
GI Severity Index	✓		✓	✓
CBCL 1.5-5	✓		✓	✓
PSI	✓		✓	✓
VABS-II	✓			✓
Mc Arthur-CDI	✓		✓	✓
GMDS-ER	✓			✓
Blood sample: LPS, Leptin, Resistin, TNF-α, IL-6, PAI-1	✓			✓
Urinary sample: Phtalates	✓		✓	✓
Fecal sample: Calprotectin	✓		✓	✓
High-density EEG registration: power, asymmetry index and coherence in the different frequency bands	✓			✓

Legend: *ADI-R* Autism Diagnostic Interview-Revised, *ADOS-2* Autism Diagnostic Observation Schedule- Second Edition, *CARS* Childhood Autism Rating Scale, *RBS-R* Repetitive Behavior Scale-Revised, *SCQ* Social Communication Questionnaire, *GI Severity Index* Gastro-Intestinal Severity Index, *CBCL 1.5-5* Child Behavior Checklist 1.5-5, *PSI* Parenting Stress Index, *VABS-II* Vineland Adaptive Behavior Scale-Second Edition, *Mc Arthur-CDI* MacArthur-Bates Communicative Development Inventories, *GMDS-ER* Griffiths Mental Development Scale-Extended Revised, *LPS* Lipopolysaccharide, *TNF–α* Tumor Necrosis Factor-α, *IL-6* Interleukin-6, *PAI–1* Plasminogen Activator Inhibitor-1, *EEG* Electroencephalography

**Table 2:** Trial registration data

Data category	Information
Primary registry and trial identifying number	ClinicalTrials.gov NCT02708901
Date of registration in primary registry	March 4, 2016
Secondary identifying numbers	GR-2011-02348280
Source(s) of monetary or material support	Sponsor: IRCCS Fondazione Stella Maris Collaborators: Ministry of Health, Italy, Istituto di Fisiologia Clinica CNR
Primary sponsor	IRCCS Fondazione Stella Maris
Secondary sponsor(s)	Collaborators: Ministry of Health, Italy; Istituto di Fisiologia Clinica CNR
Contact for public queries	Central Contact: ES, MD, PhD; Email: e.santocchi@fsm.unipi.it
Contact for scientific queries	Study Officials: ES, MD, PhD, Study Principal Investigator; IRCCS Stella Maris Foundation, Calambrone, Pisa, Italy, 56128, e.santocchi@fsm.unipi.it
Oversight	Oversight Authorities: Italy Ministry of Health
	FDA Regulated? No
	IND/IDE Protocol? No
Review Board	Approval Status: Approved
	Approval Number: 126/2014
	Board Name: Comitato Etico Pediatrico Regione Toscana
	Board Affiliation: Servizio Sanitario Regione Toscana
	Email: comitato.etico@meyer.it
	Data Monitoring?: Yes
Brief title	Gut to Brain Interaction in Autism. Role of Probiotics on Clinical, Biochemical and Neurophysiological Parameters
Official title	Gut to Brain Interaction in Autism. Role of Probiotics on Clinical, Biochemical and Neurophysiological Parameters
Countries of recruitment	Locations: Italy, IRCCS Stella Maris Foundation
Health condition(s) or problem(s) studied	Conditions: Autism Spectrum Disorder
Intervention(s)	Active comparator: Vivomixx®, Two packets (900 billions bacteria) per os (P.O.) daily x 1 month and one packet (450 billions bacteria) P.O. daily x 5 months
	Placebo comparator: Two packets (4.4 g of maltose and silicon dioxide x 2) P.O. daily x 1 month and one packet (4.4 g of maltose and silicon dioxide) P.O. daily x 5 months
Key inclusion and exclusion criteria	Eligibility: Minimum Age: 18 Months; Maximum Age: 72 Months; Gender: Both; Accepts Healthy Volunteers?: No
	Inclusion Criteria: age-range: 18–72 months; ASD diagnosis according to Diagnostic and Statistical Manual of Mental Disorders-5 (DSM-5) criteria
	Exclusion Criteria: brain anomalies detected by Magnetic Resonance Imaging (MRI); neurological syndromes or focal neurological signs; anamnesis of birth asphyxia, severe premature birth (≤28 gestational weeks) or perinatal injuries; epilepsy; significant sensory impairment; diagnosis of organic GI Disorder (i.e. gastroesophageal reflux, food allergies, Inflammatory Bowel Disease); diagnosis of Coeliac Disease; special diet (i.e. gluten-free diet, casein-free diet, high-protein diet, ketogenic diet)
Study type	Interventional
	Allocation: Randomized; Intervention Model: Parallel Assignment; Number of Arms: 4; Masking: Double Blind (Subject, Caregiver, Investigator, Outcomes Assessor)
	Primary Purpose: Treatment
	Study Phase: Phase 4
Date of first enrolment	November 2015
Target sample size	100
Recruitment status	Recruiting
Primary outcome(s)	Changes in severity level of ASD symptomatology (Time Frame: 6 months; not designated as safety issue)
Key secondary outcomes	Changes in GI symptomatology; changes in Electroencephalogram (EEG) power, coherence and asymmetry; changes in levels of serum Lipopolysaccharide, Leptin, Resistin, Tumor Necrosis Factor – α, Interleukin- 6, P Plasminogen Activator Inhibitor – 1; changes in levels of fecal calprotectin; Changes in global ASD symptomatology assessed by Childhood Autism Rating Scale, by Social Communication Questionnaire, changes in ASD symptomatology: repetitive behaviors and sensory profiles, changes in Developmental Quotient, in Adaptive Functioning, in Behavioral Profiles, and in parental stress (time frame: 3 and 6 months; not designated as safety issue).

**Table 3:** Composition, roles, and responsibilities of the research team coordinating and conducting the trial (Steering Committee), of the Auditing Committee and of the G-BIA (Gut-Brain Interaction in Autism) group

Research leading team/Steering Committee (see title page and author section for members)	Roles and Responsibilities
• Principal Investigator (Trained research child psychiatrist)	Design and conduct of the trial, preparation of protocol and revisions preparation of investigators brochure (IB) and CRFs [case report forms], organizing steering committee meetings, recruitment coordination, clinical assessment coordination, EEG recording coordination, intervention coordination, adverse events monitoring, data collection and entry coordination, providing of annual and final reports to the ethics committee and mid-time and final report to the Italian Ministry of Health, publication of study reports.
• Sub-Investigator (Trained research pharmaceutical chemical doctor)	Design and conduct of the trial, preparation of protocol and revisions, preparation of IB and CRFs, biochemical samples collection and analysis, biochemical measurement coordination, data collection and entry coordination, contacts with the company supplying probiotic and matched placebo and performing randomization and intervention assignment, coordination of statistical analysis, providing of annual and final reports to the ethics committee and mid-time and final report to the Italian Ministry of Health, publication of study reports.
• Trained research Biomedical Engineer	EEG data processing, EEG statistical analysis Contribution to the development of the study design
• Laboratory Technician	Biochemical measurement Contribution to the development of the study design
• Trained research child psychologist	Clinical assessment and data collection and entry coordination Contribution to the development of the study design
• Trained research child psychiatrist	Clinical assessment and data collection and entry coordination Contribution to the development of the study design
• Trained research child psychiatrist	Managing of the unblinding of allocated concealed treatment to families by necessity (harms or serious adverse events) - Contribution to the development of the study design
Auditing Committee (see title page and author section for members)	Roles and responsibilities
• Steering committee • Senior trained research physicians	Reviewing progress of study trough periodic audits of all the relevant aspects of the clinical trial management
G-BIA (Gut-Brain Interaction in Autism) group (see Acknowledgment section for members)	Selection of children from involved Neuropsychiatric Centers; collection of data

Clinical evaluation will include neurological and psychiatric examination along with standardized assessment of GI symptoms, autism severity, sensory-behavioral features, comorbid symptoms, parental stress, cognitive development, adaptive functioning, and language abilities.

At T0 each subject will be classified as belonging to the GI group or to the Non-Gastro Intestinal (NGI) group on the basis of the presence of significant GI symptoms measured through the Gastrointestinal Severity Index (GI Severity Index) [60]. A total score of 4 and above at the GI Severity Index (with at least 3 score points from the first six items of the scale) will be considered clinically significant for the classification of a subject within the GI group.

At T0 blood, urinary and fecal samples will be also collected from each subject in order to perform biochemical evaluation. Specifically, blood samples will be collected to measure levels of leptin, resistin and inflammatory markers: TNF- α, IL-6, LPS and Plasminogen Activator Inhibitor-1 (PAI-1); urinary samples will be collected for the analysis of urinary concentrations of primary and secondary metabolites of phthalates; fecal samples will be collected for the analysis of fecal calprotectin levels.

Neurophysiological patterns will be assessed through QEEG measures. Neurophysiological characterization will be performed by collecting EEG (Electroencephalography) data during 6–8 min of rest with eyes open. The 128-channel HydroCel Geodesic Sensor Net (HCGSN 128; Electrical Geodesics Inc., USA) system will be used for recording the EEGs. After pre-processing, QEEG techniques will be applied in order to extract relevant features characterizing brain activation and connectivity.

### Interventions

ASD participants belonging to the two groups (GI and NGI) will be blind randomized 1:1 to regular diet with probiotics or with placebo for 6 months. Therefore, four subgroups will be obtained:

- Group 1) GI symptoms and probiotics
- Group 2) GI Symptoms and placebo
- Group 3) Non-GI symptoms and probiotics
- Group 4) Non-GI symptoms and placebo

The probiotic preparation selected for this study is a mixture (Vivomixx®), each packet of which contains 450 billions of lyophilized bacterial cells belonging to eight probiotic strains: one strain of Streptococcus thermophilus DSM 24731, three strains of Bifidobacterium (B. breve DSM 24732, B. longum DSM 24736, B. infantis DSM 24737), and four strains of Lactobacillus (L. acidophilus DSM 24735, L. plantarum DSM 24730, L. paracasei DSM 24733, L. delbrueckii subsp. bulgaricus DSM 24734). Vivomixx® is a patented and marketed product and it has been approved for the use in children. This study protocol will require the oral administration of Vivomixx® which is available in a water-soluble formulation that can be dissolved directly in the mouth or in a cold, not carbonated liquid. According to the daily weight-based dose of Vivomixx® and the age and weight range of ASD children who will be recruited in the study, the protocol posology will be two packets a day (900 billions bacteria) in the first month of treatment and one packet a day (450 billions bacteria) in the following 5 months of treatment.

The treatment will be administered to children at home by their parents/legal guardian during the 6 months of the treatment protocol. A treatment duration of 6 months has been chosen in order to warrant a good colonization of the gut, that requires 2–3 weeks on average, and its maintenance for a period of time long enough to determine possible significant clinical changes.

The placebo’s packaging and organoleptic characteristics will be identical to the probiotic ones. Each packet of placebo will contain 4.4 g of maltose and silicon dioxide.

It will not be possible to modify allocated interventions for a given trial participant. Participants will discontinue interventions as a consequence of intolerable adverse events reported by parents/legal guardians or if requested by their parents/legal guardians as a free choice.

The suspension of any other treatment or intervention effective and recommended by current guidelines in ASD will not be required during the experimental protocol of treatment with probiotic/placebo.

In order to improve adherence to intervention protocols, parents/legal guardians will have the possibility of contact the research team by phone or e-mail every time they will need for assistance and information. Periodical call and direct interviews to parents/legal guardians at several time-points (Table 1) will be useful for monitoring adherence.

### Mid-time assessment

After 3 months from the enrollment (Time 1 - T1), each subject will undergo a second clinical and biochemical evaluation in order to obtain a mid-time level of some of the secondary clinical and biochemical outcome measures (Table 1). Only urinary and fecal samples will be collected in order to determine mid-time levels of urinary phthalates and fecal calprotectin.

Anamnestic information about the compliance to the protocol and possible side effects of the treatment will be specifically investigated. Clinical evaluation will include neurological and psychiatric examination along with standardized assessment of GI symptoms, autism severity, sensory-behavioral features, comorbid symptoms, parental stress, and language abilities. Detailed treatment data will be also collected.

### Final assessment

After 6 months (Time 2 - T2) from the enrollment i.e. at the end of the intervention, each subject will undergo a final assessment through the same clinical, biochemical and neurophysiological evaluations performed at T0 in order to establish the after-treatment modifications of all the primary and secondary outcome measures.

### Study design and methodologies

The study will be a double-blind randomized controlled trial with a nutritional supplement and with four parallel arms (see Table 2 for Trial Registration Data). The written informed consent from a parent or legal guardian of children will be obtained by one of the investigators involved in the recruitment, after the study procedures will be explained.

The assessment will be conducted through the administration of the golden standard diagnostic tools for ASD and through the use of psychological testing and scales currently used by scientific community for the definition of core features and comorbid symptoms of ASD. All these evaluations will be performed by a multidisciplinary research team (see Table 3) composed by a pharmaceutical chemical doctor and experienced clinically trained research child psychiatrists and child psychologists, who met standard requirements for research reliability for Autism Diagnostic Observation Schedule-2 (ADOS-2) [61] and Autism Diagnostic Interview-Revised (ADI-R) [62] (which will be described in detail in the Outcome Measures section).

Plasma and urinary levels of inflammatory markers (TNF-α, IL-6, PAI-1), leptin and resistin will be measured by Elisa immunoassay Kits using luminex Technology (Merck Millipore). Concentrations of phthalates and their metabolites will be measured in plasma and urine using LC-MS (Liquid Chromatography–Mass Spectrometry). These measurements will be performed at the molecular laboratory of Institute of Clinical Physiology of National Research Council (Pisa, Italy).

As for the analysis of brain activity and neurophysiological patterns of the recruited subjects, we will use the HCGSN 128 system. All data processing will be run offline on a computer running EEGLAB and a Lab tool for QEEG analysis. The EEG system HCGSN 128 consists of an integrated hardware/software system for recording and analyzing EEG signals from 128 channels. The presence of 128 channels distributed on the surface of the head allows obtaining in a high-density EEG recording with an inter-electrode distance of 35 mm for adults and 10 mm for children. Since the sensor net contains a bigger number of electrodes in comparison with the traditional EEG system, the representation of the distribution of electric potentials on the scalp is much more accurate. The net for the signals recording is constituted by sponges that are immersed in a saline solution to obtain the contact with the skin without abrasion. This characteristic makes the net application faster and less invasive for the subjects and make the system particularly suitable for young children.

### Assignment of interventions

After inclusion in the study and baseline assessment, participants will be assigned to the GI group or to the NGI group depending on their baseline scores at the GI Severity Index [60], as previously described. Then, participants of these two groups will blind receive a treatment with probiotics or with placebo according to a computer generating randomization sequence previously determined by the company supplying probiotic and matched placebo. The allocation ratio of intended numbers of participants will be 1:1 both in the GI and in the NGI comparison groups so that the number of children receiving probiotics or placebo will be identical respectively in the two GI and in the two NGI groups.

Randomization will be made in blocks with random sequences of independent block both in the GI and in the NGI groups; the order of interventions will be vary randomly within each block, so that the assignment blocking schedules will be unpredictable. The study will be double blind till its conclusion, so that subjects, caregivers, all research investigators and all the outcomes assessors will be blinded to treatment group assignment of all subjects till the end of data collection and analysis.

The packages containing probiotics will be indistinguishable from those containing placebo and will be enumerated progressively in the two clinical subgroups (GI: from 1 to 50; NGI from 1 to 50). The allocation sequence will be concealed and the matching between the randomization number and the contents of each package (probiotics or placebo) will be written in sealed envelopes (one for each recruited subject/randomization number) stored at the Institute of Clinical Physiology National Research Council (Pisa, Italy).

The research investigators will assign a number of treatment to each of the recruited children on the basis of their respective progressive order of enrollment inside each of the two subgroups. So, the first child enrolled in the GI subgroup will receive the treatment packages labelled “GI1”, the second child enrolled in the GI subgroup will receive the treatment packages labelled “GI2”, the first child enrolled in the NGI subgroup will receive the treatment packages labelled “NGI1” and so on.

Emergency unblinding will be permissible only in circumstances of harms or serious adverse events. The allocated intervention will be revealed to the parents/legal guardians of the subject by a research collaborator not involved in recruitment, treatment assignment or outcome assessment.

### Outcomes

We aim to evaluate the effects of administration of probiotics in preschooler with ASD with and without GI symptoms through the comparison of several clinical, behavioral and electrophysiological variables in the four subgroups identified. Only at the baseline, the ADI-R [62], a highly-structured interview considered one of the golden standard tool for diagnosis of ASD, will be administered to parents to obtain a standardized symptom profile of each child. Medical history and detailed treatment data will be also collected. As previously described, outcome variables will be assessed at three time points: at the baseline assessment (T0), after an interval of 3 months (T1), and at the end of intervention, after an interval of 6 months from the baseline assessment (T2). All the outcome variables will be assessed in both groups (GI and NGI) and in their respective subgroups in order to determine the effects of probiotics compared with placebo on gastrointestinal symptoms, autistic symptomatology, behavioral, biochemical and neurophysiological patterns (see Table 1).

Moreover, at the baseline, at T1 and at T2, the changes in nutritional status will be evaluated by weight, height and Body Mass Index (BMI) calculation, while the compliance and endurance at probiotic/placebo treatment, the concomitant drug consumption (with particular attention at antibiotic treatment) and the possible adverse events will be monitored by caregivers interview. The treatment compliance and the adverse events will be evaluated also after 15 days from T0 through a call interview. Possible changes in food habits and nutrition will be monitored through the collection and analysis of weekly food diaries that parents/caregivers will fill in during the 6 months of the treatment protocol.

In order to promote participant retention, given the long follow-up of 6 months, appointments will be scheduled in accordance with families’ availability and there will be periodical phone and e-mail contacts of parents/caregivers of patients for monitoring retention. For participants who will be “off-study” after the mid-time assessment (T1), at least a partial mid-time follow-up will be obtained from all the biochemical outcome measures and the clinical outcome measures collected at T1. For participants who will drop out before T1, only the data collected at T0 assessment will be analyzed in order to permit a baseline comparison between the total population of enrolled patients and the subset of patients completing the treatment.

### Primary outcome measure

The primary outcome is the improvement of severity level of ASD symptomatology, measured with ADOS-2 [61] which is a semi-structured assessment of communication, social interactions, play, imagination, and stereotyped or repetitive behaviors for the diagnosis of ASD with a demonstrated inter-rater reliability, test-retest reliability, and internal validity [63]. In this study, the primary outcome measures will be changes in the ADOS Calibrated Severity Scores (ADOS-CSS). The ADOS raw total scores have been already used as outcome measures in previous randomized clinical trials investigating effects of interventions in ASD [64–67]. The ADOS-CSS scores had more uniform distributions across developmental groups, were less influenced by participant demographics than raw totals; therefore, they are useful in comparing assessments across time and identifying trajectories of autism severity for clinical research [68].

### Secondary outcome measures

- Clinical outcome measures

The changes in the scores of the following scales and questionnaires will be measured during the treatment in order to evaluate the possible changes in the clinical profiles in children treated with probiotics compared with children treated with placebo (see Table 1).

*The GI Severity Index* [60] is a composite score designed by Schneider and colleagues to capture signs and symptoms of GI distress most commonly reported by parents of children with ASD. The index was constructed by adapting a modified Truelove and Witts Severity Index used for clinical trials in ulcerative colitis [69] to incorporate a total of nine variables relating to GI signs and symptoms: constipation, diarrhea, average stool consistency, stool smell, flatulence, abdominal pain, unexplained daytime irritability, nighttime awakening, and abdominal tenderness. We will collect data about the first eight variables adapting the original items in a self-report questionnaire filled-in by parents of children about the last 3 months. The variable “Abdominal tenderness” will be assessed during the physical exam performed at T0, T1 and T2 by a research physician.

*The Childhood Autism Rating Scale (CARS)* [70] consists of 15 items on socialization, communication, emotional responses, and sensory sensitivities intended to measure the presence and severity of ASD. The child is rated on each item based on the clinician’s observation of the child’s behavior throughout the evaluation as well as on the parent’s report. CARS classifies a child as having mild, moderate, or severe autism, or no autism.

*The Social Communication Questionnaire* (SCQ) [71] is a self-report parent questionnaire with a Current Form which explore ASD specific symptoms in the last 3 months through 40 yes-or-no items.

*The Child Behavior Checklist 1.5-5* (CBCL 1.5-5) [72] is a questionnaire completed by the parents intended to gather information about the child's behavior and which provides scores for seven syndrome scales (Emotionally Reactive, Anxious/Depressed, Somatic Complaints, Withdrawn, Aggressive Behavior, Attention Problems and Sleep Problems), three summary scales (i.e., Internalizing, Externalizing and Total Problems) and five DSM-Oriented scales (Affective Problems, Anxiety Problems, Pervasive Developmental Disorder Problems, Attention Deficit Hyperactivity Disorder Problems, and Oppositional Defiant Problems).

*The Sensory Profile* [73] is a self-report questionnaire which explore sensory processing in children through several items grouped by sensory processing, modulation, and behavioral and emotional responses. The nine factor groupings characterize children by their responsiveness to sensory input including Sensory seeking, Emotional reactive, Low endurance/tone, Oral sensory sensitivity, Inattention/distractibility, Poor registration, Sensory sensitivity, Sedentary, and Fine motor/perceptual.

*The Repetitive Behavior Scale-Revised* (RBS-R) [74] is a questionnaire completed by the parents informing for the presence of a broad spectrum of repetitive behaviors, and recently applied in Italian preschoolers with ASD [75]. It is composed of 43-item scale measuring the breadth of repetitive behavior in children, adolescents, and adults with ASD. It consists of six subscales that have no overlap of item content: Stereotyped Behavior, Self-injurious Behavior, Compulsive Behavior, Routine Behavior, Sameness Behavior, and Restricted Behavior.

*The Parenting Stress Index* (PSI) [76] is a self-report questionnaire designed to evaluate the degree of stress in the parent–child relationship. It yields a Total Score and three domain scores.

The *Vineland Adaptive Behavior Scale II* (VABS-II) [77] is a widely used parent interview scale that assesses adaptive functioning in the areas of communication, daily living, socialization, and motor skills, as well as yielding an adaptive behavior composite score.

Developmental level will be assessed trough *the Griffiths Mental Development Scales-Extended Revised* (GMDS-ER) [78], from which a Developmental Quotient will be obtained.

The MacArthur-Bates Communicative Development Inventories (CDIs) [79] are parent report instruments which capture important information about children's developing abilities in several domains of early language, including vocabulary comprehension, production, gesture use, and early grammar.

b) Electrophysiological outcome measures

The modification of the electrophysiological pattern at rest will be evaluated by the comparison of the QEEG features measured at T0 and T2 i.e. power, asymmetry and coherence (a measure of connectivity) computed within each characteristic frequency band of the brain.

c) Biochemical outcome measures

In order to value the effects of probiotics compared with placebo on the intestinal and systemic inflammation, LPS, leptin, resistin, TNF-α, IL- 6, PAI–1, phtalates and calprotectin will be measured before and after the treatment (see Table 1).

### Data management and confidentiality

All data will be entered both in paper Case Report Forms (CRF) and in an electronic database immediately after their collection at T0, T1 and T2. In order to protect confidentiality, CRF will be stored inside a locked archive at IRCCS Stella Maris Foundation, while electronic data will be stored in a secured file inside the server of the same institution. Numeric codes from 001 to 100 will be assigned to the recruited subjects on the basis of their respective order of recruitment in the study, and these codes will be reported in the CRF, in the electronic database, in the phials containing biological samples and in the files containing EEG recordings (“P001” for the first recruited child, “P002” for the second recruited child etc.). The matching between the personal data of the recruited subjects and their assigned numeric code will be reported in a separate secured electronic archive, along with their respective randomization code (“GI” from 1 to 50 and “NGI” from 1 to 50, as described above). Research collaborators not involved in outcome assessment will be allowed to access the locked archive and the electronic secured database in order to enter collected data, using unambiguous, standardized terminology and abbreviations to avoid misinterpretation. In order to improve the accuracy of data entry and coding, data entered in the electronic and paper sources will be periodically verified by independent researchers.

The Principal Investigator (ES) and the Sub-Investigator (LG) and the primary sponsor (IRCCS Stella Maris Foundation) will have access at the end of the study to the full trial dataset, and to the unblinding of treatment assignment. The Italian Ministry of Health can have access to the trial data anytime during or after the trial.

### Statistical analysis, power calculation and sample size

#### Sample size/power

The sample size calculation is based on primary outcome assumption in the intervention and control group. The primary outcome is the improvement of severity level of ASD symptomatology, measured with ADOS-2 which defines the existence of a responder. A recent study carried out by our group gives as an expected rate of responder equal to 62 % [80].

Sample size calculations were performed using the nQuery advisor 6.2 software, based on the primary efficacy variable. Assuming a response rate of 62 % in the placebo group and 90 % in the probiotic group, it was calculated that 38 patients per treatment arm would be sufficient to achieve 90 % power in detecting a treatment difference based on 1-tail w2 test at a significance level of 0.05. Therefore a number of 50 subjects to be enrolled per group was considered sufficient to reach the goal accounting for a certain degree of drop-out rate reported in previous studies on the effects of probiotics [34, 54–56].

**Fig. 1:**
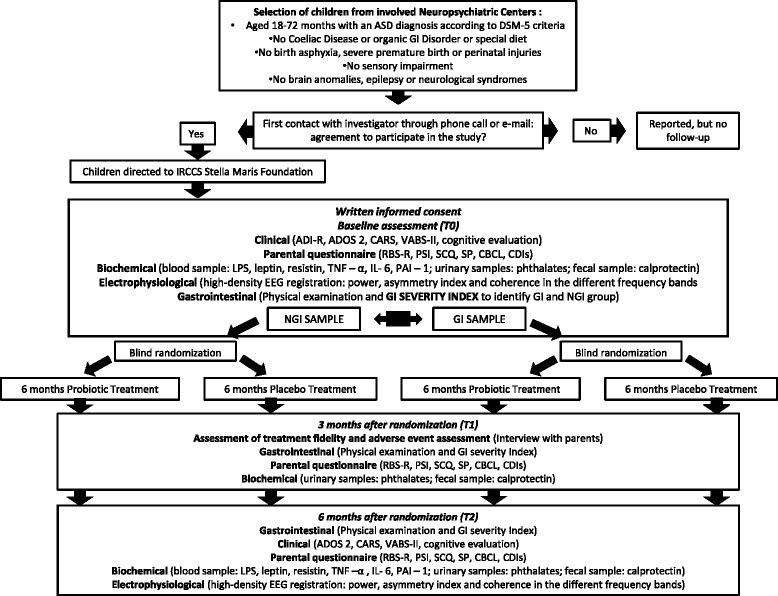
Flow-chart of the phases of the RCT. Legend: ASD: Autism Spectrum Disorders, DSM-5: Diagnostic and Statistical Manual of Mental Disorders-5th Edition, T0: Time 0, T1: Time 1, T2: Time 2, GI: Gastro Intestinal, NGI: Non-Gastro Intestinal ADI-R: Autism Diagnostic Interview-Revised, ADOS-2: Autism Diagnostic Observation Schedule- Second Edition, CARS: Childhood Autism Rating Scale, RBS-R: Repetitive Behavior Scale-Revised, SCQ: Social Communication Questionnaire, GI Severity Index: Gastro Intestinal Severity Index, CBCL 1.5-5: Child Behavior Checklist 1.5-5, PSI: Parenting Stress Index, VABS-II: Vineland Adaptive Behavior Scale-Second Edition, CDIs: MacArthur-Bates Communicative Development Inventories, GMDS-ER: Griffiths Mental Development Scale-Extended Revised, LPS: Lipopolysaccharide, TNF–α: Tumor Necrosis Factor-α, IL-6: Interleukin-6, PAI–1: Plasminogen Activator Inhibitor-1, EEG: Electroencephalography.

In order to achieve adequate participant enrolment to reach target sample size, subjects will be recruited from several clinical settings: 1) Child and Adolescence Mental Health Services of Tuscany Region (Italy), that are first level services for diagnosis and treatment of ASD, dislocated all over the region; 2) The ASD Unit of Child Psychiatry at IRCCS Stella Maris Foundation (Pisa, Italy), a third level institute who receives approximately 250 patients per year (mostly preschool children) for diagnosis from all over Italy; 3) The Unit of Child Rehabilitation at IRCCS Stella Maris Foundation (Pisa, Italy), that is a third level service for treatment of ASD and receives approximately 40 patients per year. At each clinical center involved in the study a child psychiatrist will identified potential subjects, screening them among all their patients through a specific checklist for inclusion and exclusion criteria (Fig. 1). Then the child psychiatrist will informed parents/legal guardians of selected children about the study, giving to them an advertising brochure about the research aims and design and invited them to contact the research team through phone call or e-mail in order to receive further information and to give their agreement to participate. A dedicated phone line will be available at IRCCS Stella Maris Foundation, a research assistant responds during business hours and an answering machine at all other times. All the study visits will be carefully planned and scheduled in accordance with the availability of the families in order to avoid an interference with parents’ work or with the child rehabilitation sections.

#### Data and statistical analysis

All data were will be collected and analyzed independently of the investigators, who will not have access to the data or to its analysis until the latter had been completed. Baseline and demographic data will be tested for balance between treatments using a 1-way analysis of variance (ANOVA), w2 test, or Fisher exact test, as appropriate. As designed by the protocol, the primary comparison for efficacy will be the responder rate according to ADOS 2 score; the analysis will be carried out on the intent-to-treat population, defined as all randomized patients who received at least one dose of study medication. Dropouts will be considered as no responders. Comparison between treatment groups will be carried out by Cochran/Mantel/Haenszel test for responder counts, controlled for basal value, on either the total population of enrolled patients and on the subset of patients completing the treatment. Post hoc-trend analysis of responders within each group will be carried out by the Cochran-Armitage trend test. All data will be analyzed by means of SAS 8.2 Stat Procedures.

### Data monitoring

The Italian Ministry of Health has the role of oversight and ultimate authority over all the activities of the study and requires the research team to write a mid-time report about the progress of the work and a final report about the conclusion of the research.

In the mid-time report, interim analyses will be performed on the rate of recruitment from each of the clinical settings involved, and on adherence of recruited participants to the treatment protocol. Baseline and demographic data will be tested for balance between dropouts and participants who will have completed the protocol, using a 1-way analysis of variance (ANOVA), w2 test, or Fisher exact test, as appropriate. Comparison between treatment groups will be carried out at the completion of the recruitment.

Any relevant changes to the study protocol which may impact on the conduct of the study, or significant administrative aspects need to be communicate to the Italian Ministry of Health in order to obtain a formal authorization. The Pediatric Ethic Committee of Tuscany Region has assumed a role comparable to a Data Monitoring Committee, requiring the research team to write yearly reports about the progress of the work and reports about any adverse events. Any important protocol modification during the trial need to be communicate to the Ethic Committee in order to obtain a written authorization to a protocol amendment. The Pediatric Ethic Committee of Tuscany Region is independent from the sponsor and from trial investigators. Further details about it and its charter can be found at http://www.meyer.it/index.php/ricerca-e-innovazione/comitato-etico.

Periodic audits of all the relevant aspects of the clinical trial management will be conducted by a team of senior researchers not directly involved in the trial (FM, MAM, EG). Audits will be planned every 2 months as research meetings with an order of business related to the aspects of the trial that will need a critical review (see Table 3).

### Ethical issues and consent to participate

The study protocol (version 1- 14/07/2014) has been approved by the Pediatric Ethic Committee of Tuscany Region in July 2014 (Approval Number: 126/2014). The current version is a protocol amendment (version 2- 14/11/2014) which has been approved in December 2014. The study will be carried out in accordance with recognized ethical principles and good clinical practice for clinical trials with food supplements. It will ensure the protection of individuals as recommended in the Oviedo Convention and in the Declaration of Helsinki. The suspension of any other treatment or intervention effective and recommended by current guidelines in ASD will not be required during the experimental protocol of treatment with probiotic/placebo. All the necessary information about the study protocol, including the aims, methods, potential risks and benefits related to experimentation, and their right to refuse participation or to withdraw consent at any time will be given to parents/legal guardians of children recruited for the study through an information sheet. No evaluations or other procedures will be carried out without the prior obtaining of acceptance of all the procedures described in the information sheet and signature of consent to participate by both parents/legal guardians of the subjects. Moreover, parents/legal guardians of all the children who will be recruited in the study will be asked to give a specific and separate consent to publication of data in an anonymous way.

Parents/legal guardians will be asked to signed an additional/ancillary consent for collection of additional blood, urinary and fecal samples and for their storage at −80 °C for future studies on the gut-brain connection (i.e. metabolomic analysis or bacterial culture tests).

Patients that will be enrolled into the study will be covered for non-negligent harm associated with the protocol by an appropriate insurance.

## Discussion

This randomized controlled trial will provide new information on the clinical and neurophysiological effects of probiotic treatment in preschoolers with ASD. To our knowledge, to date only four studies explored the effects of probiotics in ASD patients [34, 54–56] and they all showed several limitations related to sample size, lack of information about the probiotic oral supplementation or concurrent medications and diet [57]. Adams and colleagues [34] compared GI flora and GI status from stool samples of 58 children with ASD and 39 healthy typical children of similar ages, and found lower levels of total SCFAs in ASD group with a greater difference in children with autism taking probiotics. In that study, GI symptoms [34] were strongly correlated with the severity of autism. However, information about the type of probiotic oral supplementation was only partially explained. Kaluzna-Czaplinska [55] administered oral supplementation of Lactobacillus acidophilus twice a day for 2 months to a group of ASD children and found significant metabolic modifications, and an improvement in the ability to carry out orders and concentration, but no other behavioral or emotional effects. Parracho et al. [54] reported in a large sample of children with ASD a significant behavioral improvement in ASD treated with Lactobacillus plantarum WCSF1 compared to ASD treated with placebo. Recently, Tomova and colleagues [56] revealed a strong positive correlation of autism severity with the severity of GI dysfunction. In that study, probiotic diet supplementation (a combination of Lactobacillus, Bifidobacter, and Streptococci, three times a day for 4 months) normalized the microflora of autistic children. However, the effects of probiotic diet supplementation on behaviors were not investigated.

One of the possible outcome of this study is the identification of a subgroup of children with ASD and GI symptoms that could represent a particular endophenotype of ASD characterized by an abnormal gut microflora. We expect that these children, after a probiotic treatment, could show changes in GI symptomatology and in related blood, urinary and fecal biomarkers through their effects in restoring the balance of intestinal microflora, and in attenuating immunological abnormalities [32, 44]. Moreover, we expect that the treatment with probiotics in individuals with ASD and GI symptoms may produce also more significant improvement in autistic symptoms, in behavioral profiles, in adaptive functioning and in cognitive and linguistic development in comparison with individuals with ASD and GI symptoms treated with placebo. As far as we know, this is the first project that aims to examine the impact of this treatment not only on clinical but also on neurophysiological patterns and so it could provide new insights into the gut-brain connection in autism. According to our knowledge, only few studies on the presence of phthalates in subjects with ASD [52, 53] has been conducted. One study of 48 children with ASD and 45 control children reported that urinary concentrations of two phthalates (5-OH-MEHP [mono-(2-ethyl-5-hydroxyhexyl) 1,2-benzenedicarboxylate] and 5-oxo-MEHP [mono-(2-ethyl-5-oxohexyl) 1,2-benzenedicarboxylate]) were significantly increased in the ASD group compared with the control group [52]. Another study reported that 50 children with ASD had decreased glucuronidation of diethylhexyl phthalate, as measured by urinary metabolites, compared with 53 age-matched controls with typical development, despite similar phthalate exposure levels [53]. Notably, glucuronidation is a significant pathway involved in the metabolism of xenobiotics and lower glucuronidation might lead to a decreased detoxification capacity for phthalates. Therefore, the current project could add new data on the relationship between the presence of phthalates, clinical features and neurophysiological patterns in ASD. The expected risks for involved subjects are minimal and related to possible side effects of treatment with probiotics, which, as a dietary supplement, provides mild, transient and not hazardous side effect for the child’s health and to possible side effects related to draw blood that can be considered mild and transient, too. Nevertheless, one of the possible study limitations is the adherence of the families to the whole protocol, since it requires two blood collections, three urinary and fecal collections and two EEG registration and, even if these procedures are not very invasive. During previous analogue researches in our Institute, several families did not accept such kind of procedures for research purposes only. Moreover, adherence of the families could be limited by the blind and randomized study design, given the possibility of not obtaining immediate positive effects for their children if assigned to the placebo groups, mostly due to the relatively long duration of the treatment protocol. Another possible limitation of this study is the lack of a control sample of typically developing children with and without GI symptoms in order to obtain a comparison of the biomarkers levels, the phthalate presence and the neurophysiological findings of ASD subjects. On the basis of the protocol results, if the presence of the hypothesized endophenotype should be confirmed, a subsequent enrollment of a control matched group of children with typical development will be taken into consideration.

## Conclusions

In conclusion, the results of this project could provide a multifaceted and wide characterization of ASD, and investigate the relationship between intestinal inflammation and environmental toxicity. In this way, more solid evidence on the role of treatment with probiotics on GI function, and on behavioral and neurophysiological parameters could be obtained. At the present time, the approved and recommended treatments for ASD are mainly based on rehabilitation, educational interventions and psycho-pharmacological treatments. In children with ASD, the GI dysfunction may be associated with a higher rate of irritability, anger, aggressive behaviors and sleep disturbances [4, 7]. Therefore, the treatment of these symptoms by probiotics administration (a non-pharmacological and relatively risk-free option) could not only reduce overall costs for this disorder but also improve compliance and adherence of these individuals and of their families to educational and rehabilitation treatments.

### Trial status

Participant recruitment started in November 2015 and it is currently underway. All participants recruited up until now have been randomized and completed assessments at T0, while some of them have completed assessments at T1. The Study Completion will be in November 2017.

## Abbreviations

5-OH-MEHP, mono-(2-ethyl-5-hydroxyhexyl) 1,2-benzenedicarboxylate; 5-oxo-MEHP, mono-(2-ethyl-5-oxohexyl) 1,2-benzenedicarboxylate; ADI-R, Autism Diagnostic Interview-Revised; ADOS-2, Autism Diagnostic Observation Schedule- Second Edition; ADOS-CSS, ADOS Calibrated Severity Scores; ASD, Autism Spectrum Disorders; BMI, Body Mass Index; CARS, Childhood Autism Rating Scale; CBCL 1.5-5, Child Behavior Checklist 1.5-5; CNS, Central Nervous System; DSM, Deutsche SammLung von Mikroorganismen; DSM-5, Diagnostic and Statistical Manual of Mental Disorders-5th Edition; EEG, Electroencephalography; GI Severity Index, Gastrointestinal Severity Index; GI, gastrointestinal; GMDS-ER, Griffiths Mental Development Scale-Extended Revised; HCGSN, 128 The 128-channel HydroCel Geodesic Sensor Net; IBD, Inflammatory Bowel Disease; IL-6, Interleukin-6; LC-MS, Liquid Chromatography–Mass Spectrometry; LPS, lipopolysaccharides; Mc Arthur-CDI, MacArthur-Bates Communicative Development Inventories; NGI, Non-Gastro Intestinal; PAI-1, Plasminogen Activator Inhibitor-1; PSI, Parenting Stress Index; QEEG, Quantitative Electroencephalography; RBS-R, Repetitive Behavior Scale-Revised; SCFAs, short chain fatty acids; SCQ, Social Communication Questionnaire; T0, Time 0; T1, Time 1; T2, Time 2; TNF-α, Tumor Necrosis Factor-α; VABS-II, Vineland Adaptive Behavior Scale- Second Edition
